# GPR54-Dependent Stimulation of Luteinizing Hormone Secretion by Neurokinin B in Prepubertal Rats

**DOI:** 10.1371/journal.pone.0044344

**Published:** 2012-09-28

**Authors:** Pasha Grachev, Xiao Feng Li, Yuan Shao Lin, Ming Han Hu, Leena Elsamani, Stewart J. Paterson, Robert P. Millar, Stafford L. Lightman, Kevin T. O’Byrne

**Affiliations:** 1 Division of Women’s Health, School of Medicine, King’s College London, London, United Kingdom; 2 Department of Pharmacology & Therapeutics, School of Biomedical Sciences, King’s College London, London, United Kingdom; 3 Centre for Integrative Physiology, University of Edinburgh, Edinburgh, United Kingdom; 4 Mammal Research Institute, Department of Zoology & Entomology, University of Pretoria, Pretoria, South Africa; 5 University of Cape Town/Medical Research Council Research Group for Receptor Biology, Division of Medical Biochemistry, Faculty of Health Sciences, University of Cape Town, Cape Town, South Africa; 6 Henry Wellcome Laboratories for Integrative Neuroscience and Endocrinology, University of Bristol, Bristol, United Kingdom; University of Regensburg, Germany

## Abstract

Kisspeptin, neurokinin B (NKB) and dynorphin A (Dyn) are coexpressed within KNDy neurons that project from the hypothalamic arcuate nucleus (ARC) to GnRH neurons and numerous other hypothalamic targets. Each of the KNDy neuropeptides has been implicated in regulating pulsatile GnRH/LH secretion. In isolation, kisspeptin is generally known to stimulate, and Dyn to inhibit LH secretion. However, the NKB analog, senktide, has variously been reported to inhibit, stimulate or have no effect on LH secretion. In prepubertal mice, rats and monkeys, senktide stimulates LH secretion. Furthermore, in the monkey this effect is dependent on kisspeptin signaling through its receptor, GPR54. The present study tested the hypotheses that the stimulatory effects of NKB on LH secretion in intact rats are mediated by kisspeptin/GPR54 signaling and are independent of a Dyn tone. To test this, ovarian-intact prepubertal rats were subjected to frequent automated blood sampling before and after intracerebroventricular injections of KNDy neuropeptide analogs. Senktide robustly induced single LH pulses, while neither the GPR54 antagonist, Kp-234, nor the Dyn agonist and antagonist (U50488 and nor-BNI, respectively) had an effect on basal LH levels. However, Kp-234 potently blocked the senktide-induced LH pulses. Modulation of the Dyn tone by U50488 or nor-BNI did not affect the senktide-induced LH pulses. These data demonstrate that the stimulatory effect of NKB on LH secretion in intact female rats is dependent upon kisspeptin/GPR54 signaling, but not on Dyn signaling.

## Introduction

The secretion of GnRH is pulsatile and is controlled by the GnRH pulse generator [1], [2]. Pubertal onset in the female is characterized by increasingly frequent gonadotropin pulses, rising estradiol (E_2_) levels and reduced sensitivity to the negative feedback effects of E_2_ [3]. Novel components essential for the regulation of GnRH secretion, and thus physiologic pubertal development and fertility, were discovered through mutations in genes encoding GPR54 [4], [5], the putative receptor for kisspeptin, Neurokinin B (NKB) and its receptor (NK3R) [6]–[8]. Kisspeptin, NKB and NK3R are coexpressed within hypothalamic arcuate nucleus (ARC) neurons, which might comprise the GnRH pulse generator, along with kappa-opioid receptor (KOR) and its ligand, dynorphin A (Dyn) [9]–[11]. Expression of the genes encoding kisspeptin and its receptor (*Kiss1* and *Kiss1R*, respectively) increases at puberty [12], [13], as does NKB- and Dyn-immunoreactivity [14]. Moreover, exogenous kisspeptin stimulates precocious LH secretion and puberty in rats [15]. Current research endeavors to unravel the complex roles of kisspeptin/GPR54, NKB/NK3R and Dyn/KOR (collectively, KNDy) signaling systems, and the interaction between these, in determining the activity of the GnRH pulse generator, have employed a range of animal models.

It is widely accepted that kisspeptin mediates the stimulation of GnRH and gonadotropin secretion [16], [17], while dynorphin is involved in the suppression of gonadotropin release [18], [19]. Conversely, recent findings concerning the role and mode of action of NKB are apparently contradictory (in part due to differences in the species, sex, gonadal status and methodology used) [20]. We have recently shown that central (intracerebroventricular or intra-ARC) administration of the NKB analog, senktide, dose-dependently suppresses pulsatile LH secretion in ovariectomized (OVX) rats replaced with low levels of E_2_ [21], [22]. We have also shown that intra-ARC injection of a KOR agonist, U50488, similarly decreases LH pulse frequency, and demonstrated that the inhibitory influence of ARC NK3R activation on pulsatile LH secretion is dependent on Dyn/KOR signaling [22]. On the contrary, the presence of relatively high levels of E_2_, such as those in intact rats or in OVX rats treated with high levels of E_2_, appear to reverse the inhibitory influence of NKB/NK3R signaling, rather stimulating LH secretion [23]. Indeed, E_2_ levels have long been known to play such a modulatory role in the regulation of numerous neuropeptides [24]. Thus it appears that NKB flexibly mediates the effects of E_2_ negative feedback on the HPG axis, at least in the rat. While considerable advances have recently been made in the understanding of the roles of KNDy signaling systems in modulating pulsatile LH secretion in adults, little is currently known about the involvement of these neuropeptides in regulating LH secretion in the developing animal.

Although kisspeptin/GPR54 signaling has been considered a prerequisite for normal reproductive development and fertility, a recent study demonstrated that reproductive maturation can occur in transgenic female mice apparently devoid of kisspeptin neurons through conditional ablation [25]. However the same study also documents disrupted cyclicity and infertility in mice in which kisspeptin neurons were ablated postnatally [25]. It has recently been demonstrated that both NKB and kisspeptin analogs stimulate LH secretion in prepubertal agonadal male macaques [26]. The same study reported that desensitization of GPR54 abrogated the senktide-induced increase in LH levels [26], suggesting that NKB elicits LH secretion in a kisspeptin/GPR54 dependent fashion. The kisspeptin/GPR54-dependent nature of HPG axis stimulation by NKB has since been evidenced in male GPR54-knockout mice [27]. These findings are consistent with the notion that other stimulatory cues may compensate for the lack of kisspeptin/GPR54 signaling during pubertal development. To this end there is no consensus on the importance of kisspeptin/GPR54 signaling in reproductive development.

There is evidence for sexual dimorphism of ARC kisspeptin/NKB neurons in prepubertal mice [28] and rats [14], as well as for sex differences in the regulation of LH secretion in rodent species [28], [29]. Currently little is known about the involvement of the kisspeptin/GPR54 signaling system in mediating the effects of NKB/NK3R signaling on LH secretion in the prepubertal female. Therefore in the present study we investigated whether, like in the male monkey and the male mouse, NK3R agonism stimulates pulsatile LH secretion in the prepubertal female rat. Since presently the kisspeptin-NKB interaction is poorly understood, we also investigated whether central effects of senktide are mediated by the kisspeptin/GPR54 system. Finally, we tested the hypothesis that the inhibitory Dyn/KOR signaling system is not involved in the modulation of pulsatile LH secretion under these conditions.

## Materials and Methods

### Ethics Statement

All animal procedures were undertaken in accordance with the Animals (Scientific Procedures) Act UK, 1986, and were approved by the King’s College London Ethical Review Panel Committee. All surgical procedures were carried out under anesthesia induced by ketamine (Vetalar, 100 mg/kg, ip; Pfizer, Sandwich, UK) and xylazine (Rompun, 10 mg/kg, ip; Bayer, Leverkusen, Germany), and all efforts were made to minimize suffering.

### Animals and Surgical Procedures

Adult Sprague-Dawley rats (Charles River, Margate, UK) were allocated into breeding pairs. Litters were assessed daily, and the day of birth was considered postnatal day one (PND 1). Excess male offspring were culled to reduce litters to 10–12 pups on PND 3. Pups were weaned onto standard rat chow (RM1; SDS Diets, Witham, UK) on PND 21 and were housed 4–5 per cage under controlled conditions (12∶12 h light/dark cycle, lights on 0700 h, temperature, 22±2°C) and provided with food and water *ad libitum.*

On PND 26 female rats were fitted with a unilateral guide cannula (22 gauge; Plastics One, Roanoke, VA) directed towards the left lateral ventricle, the coordinates for implantation being 1.2 mm lateral, 1 mm posterior to Bregma, and 4.5 mm below the surface of the dura, according to the rat brain atlas of Paxinos and Watson [30]. The guide cannula was secured using dental cement (Dental Filling, Swindon, UK), and fitted with a dummy cannula (Plastics One) to maintain patency [31]. Correct cannula placement was confirmed by the observation of gravitational meniscus movement upon insertion of an internal injection cannula (Plastics One) with extension tubing preloaded with artificial cerebrospinal fluid (aCSF). At the time of icv cannulation rats were also fitted with a single indwelling cardiac catheter via the right jugular vein [32]. The catheter was exteriorized at the back of the head and enclosed within a 30-cm long lightweight metal spring tether (Instec Laboratories, Boulder, CO) secured to a cranial attachment. The distal end of the tether was attached to a fluid swivel (Instec Laboratories), allowing the rat to move freely. After surgery, animals were housed singly and allowed 2 d recovery before experimentation.

### Experimental Design

On the morning of experimentation, 1 ml normal saline (Animalcare, Dunnington, UK) was intravenously (iv) administered over 10 min and the rats were then attached via the cardiac catheters to a computer-controlled automated blood sampling system, which enables intermittent withdrawal of 30-µl blood samples without disturbing the rats [31]. Once connected, the animals were left undisturbed for 1 h before blood sampling commenced (between 1000 and 1200 h) then blood samples were taken every 5 min for 3 h. After removal of each blood sample, an equal volume of heparinized saline (50 U heparin sodium/ml normal saline; Wockhardt, Wrexham, UK) was infused. Blood samples were incubated on ice during the experiment and then frozen at −20°C.

After a period of control blood sampling an icv injection cannula with extension tubing, preloaded with drug or vehicle (aCSF), was inserted into the guide cannula. The distal end of the tubing was extended outside of the cage to allow remote infusion without disturbing the rat. In order to reduce the effects of stress caused by cannula insertion, rats were habituated to handling twice daily for 2 days. Each injection was given in a volume of 4 µl vehicle, with 2 µl air separating the treatments. Each treatment regimen was preloaded within a single injection cannula-tubing-syringe assembly. After the bleeding procedure, rats were replaced with 1.5 ml whole blood, i.v. over 15 min. A further 1 ml whole blood was administered i.v. over 10 min the following day to preserve the hematocrit and maintain blood volume. Animals were monitored each morning for vaginal opening as a marker of puberty.

Both experiments were repeated 2–4 times with a 48 h interval between repetitions (age 28–34 d), and only rats that had not undergone vaginal opening were included. Treatments were randomized in each repetition of both experiments following a crossover design, whereby each repetition featured at least one rat subjected to each of the treatment groups. If any rat was assigned to the same treatment group more than once, the mean of the replicates was analysed.

### Effect of NK3R Agonist on LH Secretion Following Pre-Treatment with GPR54 Antagonist

Infusion regimen consisted of three consecutive injections of vehicle or 2 nmol selective GPR54 antagonist, Kp-234 (Tocris, Bristol, UK), each administered over 5 min, 20 min apart, with a further injection of vehicle or 300 pmol selective NK3R agonist, senktide (Tocris), administered 15 min following the first injection. The treatment groups were as follows: Kp-234×3+ vehicle (negative control; *n* = 3), vehicle ×3+ senktide (positive control; *n* = 3), Kp-234 ×3+ senktide (*n* = 9).

### Effect of NK3R Agonist on LH Secretion Following Pre-Treatment with KOR Agonist or Antagonist

Senktide (300 pmol) or vehicle were administered 15 min following icv pre-treatment with 400 nmol KOR agonist (U50488; Tocris), or 6.8 nmol KOR antagonist (nor-BNI; Tocris), or vehicle. The treatment groups were as follows: U50488+ vehicle (negative control; *n* = 4), nor-BNI + vehicle (negative control; *n* = 4), vehicle + senktide (positive control; *n* = 5), U50488+ senktide (*n* = 9), nor-BNI + senktide (*n* = 10).

### RIA for LH Measurement

A double-antibody RIA (NIDDK, Bethesda, MD) was used to determine LH concentrations in the blood samples [33]. Referenced preparation was rLH-RP-3. The sensitivity of the assay was 0.093 ng/ml. The intra-assay variation was 7.3% and the inter-assay variation was 8.7%.

### Data Analysis

Verification of LH pulses was established using the algorithm ULTRA [34]. Two intra-assay coefficients of variation of the LH RIA were used as the reference threshold for pulse detection. The effect of treatments on LH secretion was calculated by comparing the area under the LH profile (AUC) in the 30-min period immediately following the first injection with that in the 30-min baseline (pre-treatment) period immediately before the time of injection, using SigmaPlot v.11 (Systat Software, San Jose, CA). Where a spontaneous LH pulse, defined as a pulse that did not coincide with the timing of treatments, was detected within the 30-min period immediately before the time of the first injection, the baseline period used in analysis was designated as any 30-min period within the control (pre-injection) bleeding period devoid of LH pulses. Values given in the text and figures represent mean ± SEM. Statistical significance was tested using one-way ANOVA and Duncan’s New Multiple Range post-hoc test. *P*<0.05 was considered significant.

## Results

### Effect of NK3R Agonist on LH Secretion Following Pre-Treatment with GPR54 Antagonist

**Figure 1:**
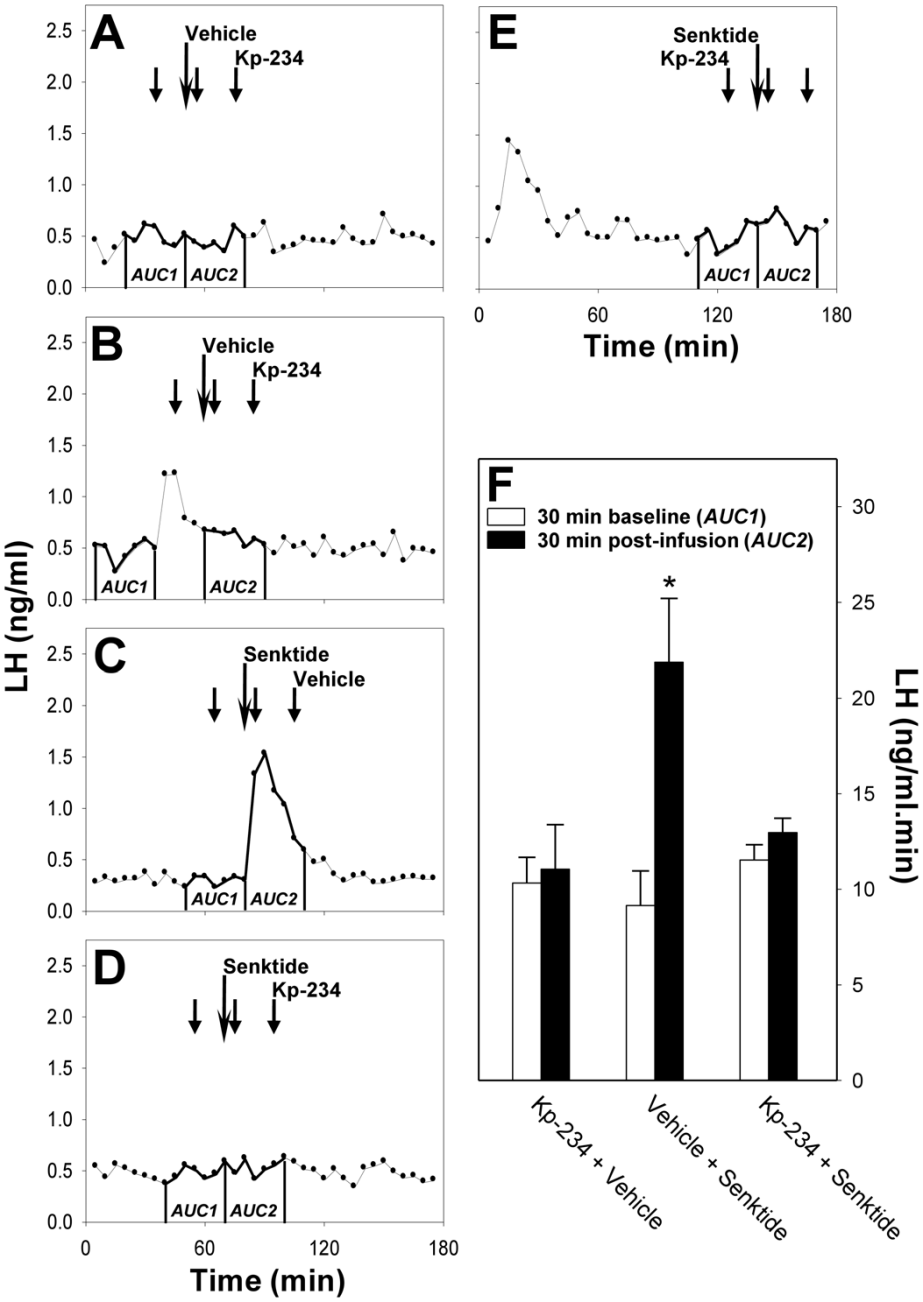
Effect of NK3R agonist on LH secretion following pre-treatment with GPR54 antagonist. Representative LH profiles demonstrating the effect of icv administration (long arrow) of a selective NK3R agonist, senktide (**C, D, E**), or vehicle (**A**, **B**) on pulsatile LH secretion in gonadal-intact prepubertal female rats in the presence (**A**, **B**, **D, E**; short arrows) or absence (**C**) of the selective GPR54 antagonist, Kp-234. Central administration of senktide induced a single LH pulse (**C**), while intermittent infusion of Kp-234 had no effect on basal LH levels (**A**, **B**). Kp-234 potently blocked senktide-induced LH pulses (**D**). Where spontaneous LH pulses (**B, E**) were detected during the pre-treatment period, these were excluded from analysis by comparing the previous 30-min period devoid of spontaneous LH pulses with the 30-min post-treatment period (**B**). Administration of Kp-234 immediately following a spontaneous LH pulse tended to attenuate the pulse (**B**), as compared to spontaneous LH pulses occurring considerably prior to Kp-234 administration, which were unaffected (**E**). The 30-min post-treatment period commenced at the time of the senktide/vehicle injection (long arrow). Area under the curve (AUC) values in the 30-min pre-treatment (baseline) period (AUC1) and the 30-min post-treatment period (AUC2) for the three treatment groups are compared in the experiment summary (**F)**. *P<0.05 versus 30-min pre-treatment (baseline) period within the same treatment group, as well as versus the same 30-min period within the group treated with Kp-234 and senktide; *n* = 3−9 per group.

In order to investigate the effect of NK3R agonism on the HPG axis and explore the interaction between the NKB/NK3R and kisspeptin/GPR54 signaling systems in the prepubertal female rat, we analysed patterns of LH secretion following icv injections of senktide in the presence and absence of Kp-234 (Fig. 1). Co-administration of Kp-234 and vehicle (Fig. 1 A, B) had no effect on LH secretion (AUC pre-treatment versus post-treatment, 10.3±1.4 ng/ml.min versus 11.1±2.3 ng/ml.min; *P*>0.05). Senktide administered with vehicle induced a single LH pulse (AUC pre-treatment versus post-treatment, 9.2±1.8 ng/ml.min versus 21.9±3.3 ng/ml.min; *P*<0.05), which coincided invariably with the timing of the senktide injection (Fig. 1 C). Spontaneous LH pulses (Fig. 1 B, E) occurred randomly. The senktide-induced LH pulse was blocked by the co-administration of Kp-234 (Fig. 1 D, E; AUC pre-treatment versus post-treatment, 11.5±0.8 ng/ml.min versus 13.0±0.8 ng/ml.min; *P*>0.05).

### Effect of NK3R Agonist on LH Secretion Following Pre-Treatment with KOR Agonist or Antagonist

**Figure 2:**
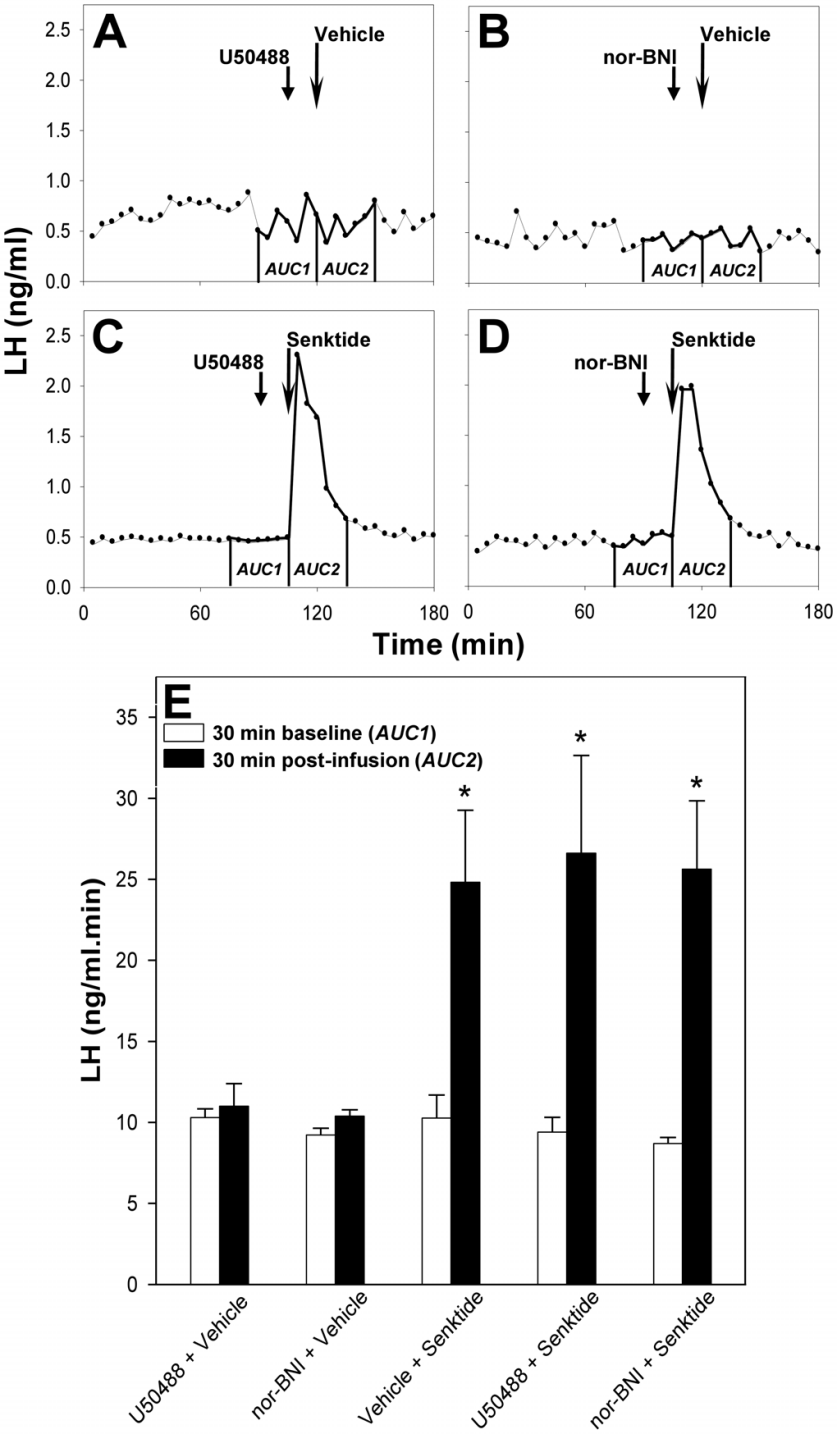
Effect of NK3R agonist on LH secretion following pre-treatment with KOR agonist or antagonist. Representative LH profiles demonstrating the effect of icv administration (long arrow) of a selective NK3R agonist, senktide (**C**, **D**), or vehicle (**A**, **B**) on pulsatile LH secretion in gonadal-intact prepubertal female rats in the presence (short arrows) of the selective KOR agonist, U50488 (**A**, **C**), or the selective KOR antagonist, nor-BNI (**B**, **D**). Central infusion of U50488 or nor-BNI had no effect on basal LH levels (**A**, **B**). Likewise, neither U50488, nor nor-BNI, affected the senktide-induced LH pulses (**C**, **D**). The 30-min post-treatment period commenced at the time of the senktide/vehicle injection (long arrow). Area under the curve (AUC) values in the 30-min pre-treatment (baseline) period (***AUC1***) and the 30-min post-treatment period (***AUC2***) for the five treatment groups are compared in the experiment summary (**E)**. *P<0.05 versus 30-min pre-treatment (baseline) period within the same treatment group, as well as versus the same 30-min period within the group treated with U50488 and vehicle, and with nor-BNI and vehicle; *n* = 4−10 per group.

To establish whether Dyn/KOR signaling is involved in the control of pulsatile LH secretion in the prepubertal female rat, and whether the Dyn/KOR and NKB/NK3R signaling systems interact in this animal model, we monitored LH secretion profiles following icv administration of senktide in rats pretreated with U50488 or nor-BNI (Fig. 2). Administered with vehicle, neither U50488 (Fig. 2 A), nor nor-BNI (Fig. 2 B), affected baseline LH levels (AUC pre-treatment versus post-treatment, 10.3±0.5 ng/ml.min versus 11.0±1.4 ng/ml.min and 9.2±0.4 ng/ml.min versus 10.3±0.4 ng/ml.min, respectively; *P*>0.05). Single LH pulses induced by senktide administration (AUC pre-treatment versus post-treatment, 10.3±1.4 ng/ml.min versus 24.8±4.4 ng/ml.min; *P*<0.05) were unaffected by pre-treatment with U50488 (Fig. 2 C; AUC pre-treatment versus post-treatment, 9.4±0.9 ng/ml.min versus 26.6±6.0 ng/ml.min; *P*<0.05) or nor-BNI (Fig 2 D; AUC pre-treatment versus post-treatment, 8.7±0.4 ng/ml.min versus 25.6±4.2 ng/ml.min; *P*<0.05).

## Discussion

This report provides evidence to indicate that selective activation of NK3R in gonadal-intact prepubertal female rats robustly elicits single LH pulses. We also demonstrate herewith that senktide-induced LH pulses are potently blocked by Kp-234, thus indicating that the stimulatory effect of NK3R agonism on LH secretion in this animal model is GPR54-dependent. These findings concur with previous data from the prepubertal male monkey [26] and adult GPR54-knockout male mouse [27] that suggest that a hierarchical functional relationship links the NKB/NK3R and kisspeptin/GPR54 signaling systems. Furthermore, our data indicate that an endogenous Dyn tone is not apparent in this animal model, that augmentation or inhibition of Dyn/KOR signaling does not alter baseline LH secretion in prepubertal female rats, and that the senktide-induced LH pulses are unaffected by neuropharmacological modulation of Dyn/KOR signaling. This is the first investigation of dynorphinergic mechanisms in the control of pulsatile LH secretion in the developing female rat, though it is in agreement with previous investigations interrogating prepubertal sensitivity to non-specific opioid blockade [35]–[41].

In the final stages of manuscript preparation another article was published documenting the stimulatory effect of icv administration of senktide on LH secretion in intact prepubertal female rats [42]. Despite some redundancy in the observations made by this and the present work, our data not only provides better resolution to the dynamics of the temporal changes in LH levels following senktide administration, but also furthers the notion of NKB-kisspeptin interaction in the generation of pulsatile gonadotropin secretion. Our results show that the senktide-induced increase in LH levels, as previously documented in this animal model [42], in fact resembles a single LH pulse that bears similarity to LH pulses arising spontaneously, which are perhaps driven by endogenous NKB/kisspeptin tones. Furthermore, a complete absence of LH pulses, senktide-induced and spontaneous, was observed when Kp-234 was administered. Additionally, there was no effect of Kp-234 on basal LH levels (as defined by the absence of spontaneous or senktide-induced pulses). We also observed that the administration of Kp-234 immediately following a spontaneous LH pulse had a tendency to decrease LH levels to baseline, thereby reducing the duration and/or the amplitude of the LH pulse, though we did not specifically investigate the effect of GPR54 antagonism on spontaneous LH pulses.

It has recently been suggested from studies in transgenic mice lacking kisspeptin neurons that kisspeptin/GPR54 signaling is not essential for pubertal development and fertility, though acute ablation of GPR54 neurons in adults disrupted physiologic cyclicity and resulted in infertility [25]. Likewise, despite severe hypogonadotropic hypogonadism, a proportion of male and female *Kiss1*- and *Kiss1r*-knockout mice exhibited spermatogenesis and vaginal estrus, respectively [43], and *Kiss1/Kiss1r* double-heterozygotes are fertile with only mild effects on reproductive function [44]. In the recent years more and more similarities between the effects of GPR54 and NK3R activation on the HPG axis in prepubertal rodents have been documented. First, LH secretion is stimulated by both senktide [42], [45] and kisspeptin [15]. Second, we present data that implicate the kisspeptin/GPR54 signaling system as a prerequisite of the stimulatory effects of NK3R activation on pulsatile LH secretion, since transient deactivation of kisspeptin/GPR54 signaling blocks senktide-induced LH secretion. Third, kisspeptin infusion advances pubertal onset [15], while antagonism of NK3R [42] or GPR54 [46] results in pubertal delay. Finally, the expression of both kisspeptin and NKB increases through peripubertal maturation [14]. These observations are consistent with the notion that kisspeptin/GPR54 signaling is not essential for pubertal development [27], [43], [44], since apparently the NKB/NK3R system might be able to compensate for its absence. Because not all ARC NKB/Dyn neurons express kisspeptin [47], conditional ablation of neurons expressing *Kiss1* should preserve a population of NKB neurons potentially capable of compensating for the lack of kisspeptin/GPR54 signaling in driving pubertal initiation and onset. Indeed, anatomical evidence from the rat shows that NKB neurons project to and form numerous close appositions with GnRH neurons [47]–[50]. However, recent evidence indicates that isolated mouse GnRH neurons are insensitive to NKB, while senktide robustly elicits firing of kisspeptin neurons [45]. Indeed, in the absence of kisspeptin neurons, other neuronal pathways may relay the stimulatory signals induced by NK3R activation to GnRH neurons [51]. Further research is necessary to establish whether kisspeptin/GPR54 signaling is indispensible for reproductive development and fertility.

In many mammalian species puberty onset is preceded by a period of insensitivity to the negative feedback effects of gonadal steroids and opioid peptides. It has therefore been postulated that endogenous opioids mediate the restraint of the HPG axis during sexual maturation. We report herewith that indeed neither augmentation, nor blockade, of Dyn/KOR signaling alters the pattern of LH secretion in ovary-intact prepubertal female rats. It has been shown that in prepubertal male monkeys [35], prepubertal gilts [36], female rabbits in the late prepubertal stage [40], as well as in prepubertal female rats [41], but not in prepubertal ewes [52], [53], that sensitivity to the non-selective opioid antagonist, naloxone, which has stimulatory effects on LH secretion in younger juveniles and adult animals, diminishes upon approach of pubertal onset. This is also apparent in prepubescent boys [37], [38] and girls [38], although there is evidence that μ-opioid receptor signaling is responsible for this phenomenon [39]. Our data support these findings.

Our results indicate that LH pulses evoked by central NK3R activation are not affected by the modulation of Dyn/KOR signaling. This observation contrasts with the notion that in adult OVX rats senktide-induced suppression of pulsatile LH secretion is Dyn/KOR-dependent [21], [22]. However, since the effects of KOR activation on LH secretion are invariably inhibitory, it is of little surprise that such a signaling system is not involved in senktide-induced LH pulse generation. It is highly plausible that diminished sensitivity to U50488, probably as a result of decreased KOR expression and density, underlies the apparent ineffectiveness of pharmacological modulators of Dyn/KOR signaling in interacting with the LH response to senktide administration.
